# Correction: Base pairing involving artificial bases *in vitro* and *in vivo*

**DOI:** 10.1039/c5sc90070k

**Published:** 2015-12-11

**Authors:** Omprakash Bande, Darren Braddick, Stefano Agnello, Miyeon Jang, Valérie Pezo, Guy Schepers, Jef Rozenski, Eveline Lescrinier, Philippe Marlière, Piet Herdewijn

**Affiliations:** a iSSB – CNRS FRE3561 , University of Evry-Val-d'Essonne , 5 rue Henri Desbruères, Genopole Campus 1, Bât. 6 , F-91030 Évry Cedex , France . Email: piet.herdewijn@rega.kuleuven.be ; Tel: +32 16 337387; b Medicinal Chemistry , Rega Institute for Medical Research , KU Leuven , Minderbroedersstraat 10 , 3000 Leuven , Belgium

## Abstract

Correction for ‘Base pairing involving artificial bases *in vitro* and *in vivo*’ by Omprakash Bande *et al.*, *Chem. Sci.*, 2016, DOI: 10.1039/c5sc03474d.



## 

In this manuscript, the captions of Fig. 10 and Fig. 11 were mistakenly swapped. The correct legends should read:

**Fig. 10** The *in vivo* interpretation of the 8-Oxo-dI nucleotide within the essential thyA gene. The normalized ratio is the experimentally derived average number of thymidine-prototrophic colonies (bla^+^ thyA^+^) from the average total number of colonies (bla^+^ thyA^–^ and bla^+^ thyA^+^). The modified section of the oligomer sequence indicates the position of the 8-Oxo-dI nucleotide/s, for single (left) and double (right) codons.

**Fig. 11** The *in vivo* interpretation of the 8-Oxo-dG nucleotide (top) and 8-Oxo-dA nucleotide (bottom) within the essential thyA gene. The normalized ratio is the experimentally derived average number of thymidine-prototrophic colonies (bla^+^ thyA^+^) from the average total number of colonies (bla^+^ thyA^–^ and bla^+^ thyA^+^). The modified section of the oligomer sequence indicates the position of the 8-Oxo-dG and 8-Oxo-dA nucleotide/s.

The Royal Society of Chemistry apologises for these errors and any consequent inconvenience to authors and readers.

